# Belladonna1Read at a meeting of the Veterinary Medical Society of the Ontario Veterinary College—session 1894-95.

**Published:** 1895-07

**Authors:** S. P. Bishop

**Affiliations:** Veterinary Student, Greencastle, Pa.


### BELLADONNA.[1]

BY S. P. BISHOP,

VETERINARY STUDENT, GREENCASTLE, PA.

In all the great field of medicine there is, perhaps, no drug which possess such a variety of actions as belladonna. Perhaps I ought to add such a variety of definite and decidedly characteristic actions.

Allow me to note at the beginning some of the physical phenomena of the plant which has been so appropriately called "Deadly Nightshade." It belongs to the order *Atropacea*, and grows in the wild state in Great Britain, and enjoys its growth uninterfered with, about hedges, margins of plantations, and in shady and deserted spots. A peculiarity about this plant is its mode of bearing leaves, which below are alternate, while higher up, or nearing its terminal point, the leaves stand out in pairs on the stock, yet while in pairs the leaves may be of unequal size and have a very short petiole. These leaves are smooth, and, though resembling hyoscyamus and stramonium, are distinguishable from them in that the leaves of hyoscyamus are hairy, while those of stramonium are much wrinkled. At no time does this plant exhibit so much activity as in June, when the flowering is complete and before the seeds are developed. While

[1] Read at a meeting of the Veterinary Medical Society of the Ontario Veterinary College—session 1894–95.

discussing its activity I might add that the plant grown by home cultivation is quite as active as that found "wild." Here, too, is seen in this respect a typical characteristic as compared with some other plants. Besides making use of the leaves and branches we utilize the roots, which should be carefully dried, and may be secured in Germany as well as in Great Britain.

In this interesting plant are found two alkaloids, atropine and belladonine, the first of which is the only one noteworthy. The both exist as true malates in the plant, and are obtained chiefly from the root by a complicated process.

Atropine when split up chemically is found to consist of tropine and tropic acid, which two, by a synthetic construction, constitute atropine again. Atropine in many respects is physiologically antagonized by morphine, eserine, and strychnine, and should never be prescribed with caustic alkalies, as they are prone to bring about its decomposition.

Many, or, I might say, most drugs possess four well-defined actions on the system, first, an immediate local action; second, an action on or in the blood; third, a specific action, and, lastly, a remote local action; but so much cannot be said about belladonna, as, although it enters the blood, it soon seeks and enters the tissues, producing little or no effect on the blood in its passage through it.

Having thus noted the physical appearance of this most interesting plant, its mode of growth, its medicinal properties, etc., let us now proceed to analyze and systematize its multiplicity of actions on the animal when administered either in medicinal or lethal doses. Belladonna or its alkaloid, atropine, is not absorbed by the unbroken skin, even though suspended in water, but when in combination with chloroform, alcohol or camphor, it is readily conducted by them through the epidermis.

We shall now proceed to administer it locally as well as internally, and note its effects and how these effects are brought about. When applied to the skin it at once depresses the sensory nerve-ending and the bloodvessels are ultimately relaxed though momentarily contracted, thus acting as a local anæsthetic.

Next proceed to the eye and drop therein some atropine, and immediately it paralyzes the endings of the third nerve, which supplies the sphincter of the pupil, and dilatation results, because the opposing sympathetic nerve is unopposed, and the radiating fibres of the iris consequently contract vigorously. Some also claim that the sympathetic may be slightly stimulated, but it is hard to believe that this drug is so specialized in its action. Thus we see that vision is wonderfully interfered with, long-sighted animals suffering most. Some contend, also, that intra-ocular pressure is diminished, but just the opposite state of affairs exists provided the dose be large.

Having briefly dealt with its action on the eye, we next apply an ointment to the mammary gland, and paralysis of the lacteal nerve termination results, and the arrest of the secretion of milk, if present, follows.

Application to the skin in a proper medium at once depresses the sudoriferous nerves, and anhydrosis follows; even a rash may make its appearance.

Do not tire in your prosecution of the phenomena of this drug, but administer internally and note its marvellous and varied effects. In the mouth we note dryness and dysphagia after its administration, as the result of paralysis of the secretory fibres of the chorda tympani nerve, which checks the secretion of saliva. The respiratory, cardiac, and vasomotor centres are much effected as we shall see. The respiratory centre is powerfully stimulated, and thus the chest movements are increased, and here perhaps is a peculiarity which ought to be noted as well as impressed; first, because herein belladonna departs from its usual line of action, and because this indicates its superiority over opium in relief of cough, spasm of bronchi, etc., which drug tends to paralyze the respiratory centre; but do not forget, however, that a poisonous dose of belladonna will also paralyze this centre. From the beginning you have no doubt observed that belladonna produced its effects by paralysis, and as we proceed you shall see the same mode of action; but on the respiratory centre does it depart from this mode, and instead of paralyzing, stimulate. Of course in the two other cases it stimulates as on the cardiac and vasomotor centres, but such stimulation is very transitory, and its effects are produced by the rapidly superseding paralysis of these centres.

In the bowel it greatly increases peristalsis by paralyzing the inhibitory branches of the splanchnics, the inhibitors of the bowel, thus leaving the unopposed accelerators to act.

On the brain we have somnolence and cerebral depression and the irritability of the cord is ultimately diminished.

On review we see the secretions in most instances are lessened, but the urine is increased, though a lethal dose may be characterized by a desire and inability to urinate. The urea, water and sulphates are increased, but the chlorides are not.

Regarding the temperature, we find it rises at first, but afterwards falls, but under small doses there is generally a rise of temperature, though large doses certainly lower it.

The irritability of motor nerves is much diminished under this drug, but the voluntary muscles remain unaffected.

Nutrition is increased by belladonna, evidently through the increase in the circulation and respiration, and thus a rise of temperature, but it falls as the circulation fails after large doses.

We have already seen that it is a sedative on the brain, consequently its administration in delirium of fevers is decidedly common-sense. Just here we might, with propriety, institute a comparison between this drug and opium, with which it is allied in some respects, though the distinctions between the two are various.

Belladonna again exerts its paralysis and the inhibitory ganglion of the heart suffers this paralysis, and acceleration results, while opium slows the heart.

The respiratory centre is stimulated by atropine, while morphine depresses it, and lastly atropine dilates the pupil while morphine contracts it.

Broad and liberal knowledge, as well as research, would certainly add much to this dissertation, but just here I must leave this interesting subject, and shali be pleased if I have in some measure thrown any light upon its properties or aided you in any way to give it its proper place in the great world of medicine.

---

Mules and oxen can work much longer without food or water than horses, and can subsist under harder work on less food, coarser provender, than the horse.

An elephant arrives at working age at twenty years and lasts until he is eighty. As much as 1200 pounds can be placed on his back for conveyance. When used for army purposes as a beast of burden, his ration usually consists of twenty-five pounds of wheat flour mixed with one pound of molasses, together with several hundred pounds of hay, green food, oftentimes sugar-cane. Thirty gallons of water are consumed by an average sized elephant in twenty-four hours.

